# Short-term cardiovascular effects and puffing behavior of exclusive heated tobacco product (HTP) users: A quasi-experimental study

**DOI:** 10.18332/tid/222366

**Published:** 2026-07-20

**Authors:** Michele Davigo, Marco Scala, Alessia Turina, Alessandra Lugo, Elisa Martelli, Frederik-Jan van Schooten, Enrico Davoli, Antoon Opperhuizen, Alexander H.V. Remels, Reinskje Talhout, Silvano Gallus

**Affiliations:** 1 Institute of Nutrition and Translational Research in Metabolism (NUTRIM), Department of Pharmacology and Toxicology, Maastricht University Medical Center+, Maastricht, the Netherlands; 2 Centre for Health Protection, National Institute for Public Health and the Environment (RIVM), Bilthoven, the Netherlands; 3 Department of Medical Epidemiology, Istituto di Ricerche Farmacologiche Mario Negri (IRCCS), Milan, Italy; 4 Department of Environmental Health Sciences, Istituto di Ricerche Farmacologiche Mario Negri (IRCCS), Milan, Italy; 5 Office of Risk Assessment and Research, Netherlands Food and Consumer Product Safety Authority (NVWA), Utrecht, the Netherlands

**Keywords:** HTPs, IQOS, cigarette, blood pressure, heart rate, puffing behavior

## Abstract

**INTRODUCTION:**

Heated tobacco products are often marketed and perceived as less harmful than regular cigarettes, although their acute cardiovascular toxicity is unknown. In this study, the acute effect of exclusive IQOS use on several cardiovascular parameters was assessed, and compared with those of both exclusive cigarette smokers and never smokers of tobacco products. The puffing behavior of both groups of tobacco users was also characterized.

**METHODS:**

An independent quasi-experimental study was conducted in Milan, Italy, in March–June 2024. Three groups of participants – frequency matched by age and gender – were recruited: exclusive conventional cigarette smokers (n=25, CS), exclusive IQOS users (n=26, IU), and never smokers (n=27, NS). All participants were aged ≥18 years, had a body mass index between 18.5 and 25 kg/m2, did not have respiratory or cardiovascular diseases, nor were they taking medications associated with these. The acute cardiovascular impact associated with the use of a regular cigarette or IQOS was measured by comparing systolic and diastolic blood pressure, and heart rate prior to (T0) and upon (T1) product consumption. Video-assisted measurement of the total length of the smoking session, number of puffs, puff duration, and inter-puff interval was carried out.

**RESULTS:**

The mean heart beats per minute of conventional cigarette (87.4 at T1 vs 74.6 at T0) and IQOS users (86.7 at T1 vs 75.5 at T0) upon product use were higher compared with their baseline measurement (p<0.001) and with the control group (70.5 at T1 vs 74.1 at T0; p<0.001). The increases (T1–T0) in the three cardiovascular measures observed for IQOS users were not different with those of conventional cigarette smokers and significantly higher than the changes of never smokers (Δ systolic blood pressure: -5.9 in NS, 2.4 in CS and 0.3 in IU; Δ diastolic blood pressure: -1.3 for NS, 4.6 for CS and 3.1 for IU; Δ heart rate: -3.7 for NS, 12.8 for CS and 11.1 for IU). IQOS users exhibited shorter smoking sessions than cigarette smokers (4 vs 3.5 minutes, p<0.05), in which they took more frequent (16.2 s for CS, 11.8 s for IU, p<0.001) and longer puffs (1.7 s for CS, 2.3 s for IU, p<0.001).

**CONCLUSIONS:**

This study shows analogous acute physiological changes of conventional and heated tobacco products on the human heart. The reported differences in puffing behaviors between cigarette and IQOS users show, to our knowledge, for the first time, the importance of using real-life puffing behavior data in laboratory settings.

## INTRODUCTION

Smoking is a major risk factor for developing coronary heart disease, stroke, aortic aneurysm, and peripheral artery disease (PAD)^1^. Approximately one-third of the estimated 8.7 million smoking-related deaths per year are attributed to cardiovascular diseases^2^. Increased awareness of the risks associated with tobacco smoke led to the implementation of anti-smoking policies, prompting the tobacco industry to develop new products, such as heated tobacco products (HTPs), marketed as less harmful than conventional cigarettes^3^. However, HTPs emissions are known to contain several well-known tobacco smoke-associated toxicants, and current literature provides contradictory findings on the relative risk of HTP use compared to conventional tobacco products^4^.

*In vivo* and *in vitro* studies comparing the cardiovascular effects of HTPs with those of conventional cigarettes conducted or funded by the tobacco industry generally suggest that switching to HTPs may lead to less detrimental impact on parameters of cardiovascular functionality (e.g. systolic and diastolic blood pressure and heart rate)^5-7^. However, independent literature reports more critical findings regarding the effects of HTPs on parameters of cardiovascular activity, suggesting that HTP use may cause arrhythmias, arterial stiffness, and cardiovascular disease with a similar magnitude of risk as conventional cigarettes^4,8-11^.

At least six industry-independent intervention studies based on 17 to 40 conventional tobacco users have been conducted to evaluate the acute effects of HTP use on selected cardiovascular outcomes in humans. All of these studies described significant alterations in these parameters, including those related to arterial stiffness, heart rate, diastolic and systolic blood pressure, after the use of HTPs^12-17^. A cross-over randomized trial on 20 conventional cigarette smokers found a significant increase in systolic and diastolic blood pressure after HTP use, but to a less extent compared to conventional cigarettes^17^. Belkin et al.^12^ used a randomized crossover trial based on 40 cigarette smokers to show that HTPs use increased primary biomarkers (high-sensitivity C-reactive protein and white blood cell count) similarly to conventional cigarettes, and resulted in endothelial dysfunction and arterial stiffness to a less extent than cigarette smoke. A study by Ioakeimidis et al.^14^ on 22 current conventional cigarette smokers showed that both HTP and conventional cigarette use were associated with increases in heart rate, blood pressure, and arterial stiffness. A similar study by Franzen et al.^15^ on 20 smokers found significant increases in systolic blood pressure and heart rate after using either IQOS or conventional cigarettes. A study on 17 occasional smokers by Goebel et al.^16^ found that HTP use leads to an acute increase in arterial stiffness and cardiovascular stress, with a substantial increase in heart rate and systolic blood pressure after use. These results suggest that use of HTPs can acutely increase arterial stiffness, cardiovascular stress, and risk factors for atherosclerosis, with most of the evidence indicating highly similar effects to those of conventional cigarettes. To the best of our knowledge, none of the available studies considered a comparison with non-users/non-smokers, nor did they differentiate between exclusive HTP users and exclusive cigarette smokers^12-17^.

Given the increasing popularity of HTPs and claims of reduced risk compared to conventional cigarettes, there is an urgent need for more industry-independent studies measuring the acute cardiovascular effects of HTP emissions in exclusive users of these products. Therefore, the Acute Health Impact of IQOS (AHIQOS) study was conducted, which aimed to assess the acute effect of exclusive IQOS use on several cardiovascular parameters, and to compare these effects with those of both exclusive cigarette smokers and never smokers of tobacco products. The study was conducted in Italy with one of the highest rates of HTP use, and the first European country where this HTP was launched^18^. Moreover, in this study, the puffing behavior of exclusive HTP users and exclusive cigarette smokers was characterized.

## METHODS

### Study design

This study was performed in the metropolitan city of Milan, Italy, in the period March–June 2024. This investigation was initially described as an observational study, as participants were free to smoke or use HTPs at their discretion. However, it could also be considered a quasi-experimental study, since participants were indirectly prompted to smoke when the research team was ready to proceed with measurements. The study was coordinated by the Mario Negri Institute (Milan, Italy), the Dutch National Institute for Public Health and the Environment (RIVM, The Netherlands), and Maastricht University (The Netherlands). The study protocol was approved by the Ethics Committee of Mario Negri Institute (Ethics Committee of Fondazione IRCCS Istituto Neurologico Carlo Besta, ID: 15, date: 17 May 2023).

Within this study, three groups of adult participants (i.e. aged ≥18 years) were recruited: 1) never smokers (NS, i.e. people who never smoked cigarettes, electronic cigarettes, or HTPs); 2) exclusive users of conventional cigarettes (CS, i.e. manufactured or roll-your-own [RYO] cigarettes); and 3) exclusive users of the heated tobacco product IQOS (IU). Subjects of the three groups were frequency-matched according to gender (male, female) and age group (i.e. 18–29, 30–44, 45–54 years).

### Recruitment and eligibility criteria

Potentially eligible study participants were approached via email, social media, and through flyers. They were asked to fill in a survey focused on their demographic characteristics and on their use of conventional cigarettes, electronic cigarettes, and HTPs. Furthermore, information regarding their general health status and self-reported diagnosis of main respiratory, cardiovascular, and metabolic conditions was collected. These data were used to assess the eligibility of each candidate.

Inclusion and exclusion criteria for each of the three study groups are summarized in Supplementary file Table 1. Never smokers were considered eligible if they never used conventional cigarettes, electronic cigarettes, or HTPs. Conventional cigarette smokers were considered eligible if they smoked ≥4 conventional manufactured or roll-your-own (RYO) cigarettes per day for ≥1 year. IQOS users were eligible when consuming ≥4 IQOS sticks (either HEETS for IQOS or TEREA for IQOS ILUMA) per day for at least 6 months, without consuming any other tobacco or nicotine product. Inclusion criteria common to all three study groups included the absence of the following health conditions: cancer, smoking-related respiratory diseases (e.g. chronic obstructive pulmonary disease), metabolic diseases, cardiovascular risk factors or cardiovascular disease (e.g. high blood pressure, high heart rate), aspects of metabolic syndrome, and use of medication associated with cardiovascular disease (e.g. blood pressure lowering medication, β-blockers, cholesterol-lowering medication). Moreover, participants with a body mass index (BMI, kg/m^2^) <18.5 (underweight) or >25.0 (overweight) were not eligible for participation in this study. Finally, pregnant or lactating women were also excluded from the study. Eligible subjects were assigned to one of the three study groups, depending on their current smoking or HTP use status. Before taking part in the study, each participant provided written informed consent and received information regarding data confidentiality and treatment of data.

### Questionnaire and measurements

Eligible participants were welcomed at the facilities of the Mario Negri Institute, read the information sheet, and were asked to sign the consent form. Subsequently, all participants completed a baseline questionnaire, in which they were asked to fill in their demographic characteristics (i.e. age and gender) and lifestyle habits (i.e. alcohol consumption). Moreover, detailed information on the use of conventional cigarettes (both manufactured and RYO), use of HTPs, and use of electronic cigarettes was obtained.

Parameters of cardiovascular functionality were measured for all the participants at baseline (T0, i.e. immediately after completing the questionnaire). After baseline measurements, CS and IU were informed that they had the possibility to consume their tobacco product in a designated outdoor area of the Mario Negri Institute at their discretion. During the smoking session, the puffing behavior of both cigarette smokers and HTP users was recorded via video to ensure precise documentation of their puffing parameters. Upon the end of the smoking session, parameters of cardiovascular functionality were measured at T1 (i.e. immediately at the end of the smoking session for cigarette smokers and HTP users, or ten minutes after the previous measurements for the group of never users). At the end of the T1 measurements, participants were thanked for their participation and were free to leave the study facilities.

### Participant compensation

All participants (independently from the study cohort) were informed prior to taking part in the study that their participation would be acknowledged with a €20 Amazon gift card. All participants included in the study received an Amazon gift card upon participation.

### Cardiovascular functionality

Prior to participation, all participants were instructed to abstain from using any nicotine products and from consuming food for at least one hour. This was the only restriction applied. Time of day was also recorded, but it was not included as a variable in the analyses. Upon arrival at the study facilities, participants from all three study groups underwent a 5-minute resting period before baseline cardiovascular measurements. All cardiovascular measurements were performed by a trained researcher, and always by the same person for standardization. Cardiovascular measures included assessment of systolic and diastolic blood pressure (mmHg) and heart frequency (beats per minutes, bpm). These measurements were collected from the left arm supported at heart level of each participant using a commercially available automatic upper arm blood pressure monitor (OMRON), with the participants sitting on a back-supported chair and feet on the floor. These indices of cardiovascular activity were measured at T0 and T1, and one measurement per time-point was performed for all participants.

### Puffing behavior

Exclusive cigarette smokers and exclusive IQOS users were allowed to consume their tobacco product in silence and alone, as they would normally do in their routine. They were sitting in a dedicated outdoor study facility, under a gazebo specifically set up for the study.

A commercially available video camera (LOGITECH) was used to film participants during the smoking session, and all participants were informed about the video recording for puffing behavior analysis and expressed their consent about it. The recording was used to determine the total length of the smoking session (minutes) and the number of puffs. The smoking session started when the first puff was taken and was terminated when the cigarette or IQOS stick was taken out of the mouth to be discarded.

The puff duration (seconds) and inter-puff interval (i.e. puff frequency, seconds) of each puff were determined by analyzing the videos using a stopwatch. All analyses were performed retrospectively. The stopwatch was activated each time a puff was initiated (e.g. when a participant placed the tobacco product in the mouth and started to puff) and stopped after the puff was taken (e.g. when the tobacco product was removed from the mouth), to record both puff duration and inter-puff interval. This analysis was performed by three independent assessors, who analyzed all video recordings and reported the total length of each smoking session, the number of puffs, the duration of each puff, and all inter-puff intervals.

### Sample size

The sample size calculation was performed using the primary research aim of the increase in heart rate after IQOS use. By using data from a previous study, a sample size of n=25 per group was determined^19^. Such a sample size allowed for the detection of a mean difference of 7.4 bpm for IQOS users compared to controls with α=0.05 and power=0.8^19^.

### Statistical analysis

GraphPad Prism 8.0 software (GraphPad Software, Boston, Massachusetts, USA) was used to carry out statistical analyses and plot the data in graphs. Two-way ANOVAs were used to compare each cardiovascular parameter (i.e. systolic blood pressure, diastolic blood pressure, heart rate) within each study group (T1 vs T0), as well as between study groups at T0 and T1. Unpaired, two-tailed t-tests were employed to examine the mean differences of the measures collected at T1 versus T0 between study groups.

Unpaired, two-tailed t-tests were also applied to compare the four puffing behavior parameters (i.e. length of the smoking session, total number of puffs, puff duration, and puff frequency) between exclusive cigarette smokers and exclusive IQOS users, and statistical significance was set at p<0.05.

## RESULTS

Table 1 shows the characteristics of the 78 participants included in the study (27 NS, 25 CS, and 26 IU), matched for gender and age; 57.7% of participants were males and 42.3% were females. In each group, the mean age was between 25.2 and 26.1 years, and the average BMI (kg/m^2^) was between 21.2 and 21.6. 28 participants announced monthly alcohol consumption: 43 participants had 1–6 alcohol units per week, and only 5 individuals reported daily alcohol consumption. Regarding tobacco use, 70% of CS and 50% of IU reported consuming <10 tobacco units (cigarettes or IQOS sticks) per day, whereas 7 CS and 13 IU reported between 10 and 15 or more units per day. Smokers of both manufactured and RYO cigarettes were eligible, but within the study, all cigarette smokers used a manufactured cigarette.

**Table 1:** Demographic and behavioral characteristics of study participants by group, Milan, Italy, March–June 2024 (N=78)

*Characteristics*	*Never smokers n (%)*	*Exclusive cigarette smokers n (%)*	*Exclusive IQOS users n (%)*
**Total,** n	27	25	26
**Sex**			
Male	16 (59.3)	14 (56.0)	15 (57.7)
Female	11 (40.7)	11 (44.0)	11 (42.3)
**Age** (years), mean (range)	26.1 (21–45)	25.2 (19–47)	25.8 (19–45)
**BMI** (kg/m^2^), mean (range)	21.2 (18.5–25.0)	21.4 (18.5–25.0)	21.6 (18.5–25.0)
**Alcohol use^a^**			
None	1 (3.7)	0 (0.0)	1 (3.9)
1–3 units per month	18 (66.7)	7 (28.0)	3 (11.5)
1–6 units per week	8 (29.6)	13 (52.0)	22 (84.6)
1 units per day	0 (0.0)	4 (16.0)	0 (0.0)
2 units per day	0 (0.0)	1 (4.0)	0 (0.0)
**Tobacco cigarettes/sticks per day^b^**			
<10		17 (70.8)	13 (50.0)
10–14		2 (8.3)	6 (23.1)
≥15		5 (20.8)	7 (26.9)

BMI: body mass index.

a One unit of alcoholic beverage corresponds to one can of beer (330 mL) or one glass of wine (125 mL) or one glass of aperitif (80 mL) or one shot glass of spirit (40 mL).

b Number of conventional manufactured and roll-your own cigarettes per day for cigarette smokers, number of IQOS sticks (HEETS or TEREA) per day for IQOS users.

**Figure 1:**
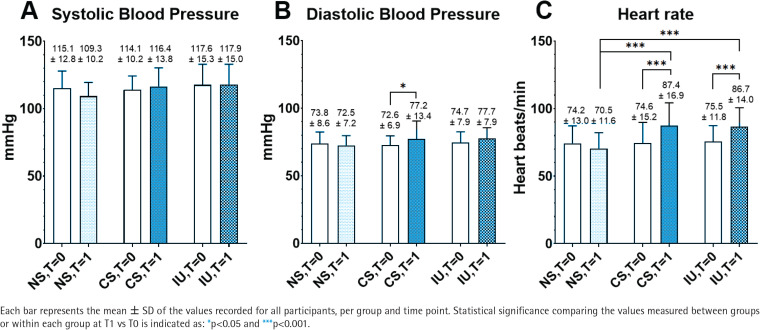
Parameters of cardiovascular functionality assessed in all participants (N=78) at T0 and T1: A) Systolic blood pressure; B: Diastolic blood pressure; and C) Heart rate (NS: never smokers, N=27; CS: exclusive cigarette smokers, N=25; and IU: exclusive IQOS users, N=26)

While no differences in systolic blood pressure were detected between study groups and time points, a significant increase in diastolic blood pressure (p<0.05) was detected only in the CS group upon product use, as shown in Figure 1B. In addition, the heart rate of both cigarette smokers and IQOS users was substantially elevated upon product use compared with never users (p<0.001) and was significantly higher than the measures obtained at T0, prior to product consumption (p<0.001) (Figure 1C).

Between T0 and T1, systolic blood pressure, diastolic blood pressure, and heart rate decreased in never smokers (-5.9 mmHg, -1.3 mmHg, -3.7 bpm, respectively) and increased in cigarette smokers (2.4 mmHg, 4.6 mmHg, 12.8 bpm) and in IQOS users (0.3 mmHg, 3.1 mmHg, 11.1 bpm) (Table 2). Changes in systolic blood pressure and heart rate significantly differed between cigarette smokers and never smokers (p<0.001) (Table 2). Changes in all assessed cardiovascular parameters were substantially different between IQOS users and never smokers (p<0.01), and no differences between the two groups of users were identified.

**Table 2:** Differences between T1 (after product consumption, or after 10 minutes for never smokers) and T0 (baseline) of cardiovascular measures of 27 never smokers, 25 exclusive smokers of conventional cigarettes and 26 exclusive IQOS users, Milan, Italy, March–June 2024

*Measures*	*Never smokers (NS)* *mean (SD)*	*Exclusive cigarette smokers (CS)* *mean (SD)*	*Exclusive IQOS users (IU)* *mean (SD)*	*CS vs NS p^a^*	*IU vs NS p^a^*	*IU vs CS p^a^*
Systolic blood pressure (mmHg)	-5.9 (6.9)	2.4 (6.6)	0.3 (8.7)	**<0.001**	**0.006**	0.349
Diastolic blood pressure (mmHg)	-1.3 (4.8)	4.6 (13.7)	3.1 (6.6)	0.052	**0.008**	0.628
Heart rate (bpm)	-3.7 (6.1)	12.8 (10.2)	11.1 (8.8)	**<0.001**	**<0.001**	0.529

a p-value of the t-test between T1 and T0. bpm: beats per minute. Estimates in bold are statistically significant at 0.05 level.

**Figure 2:**
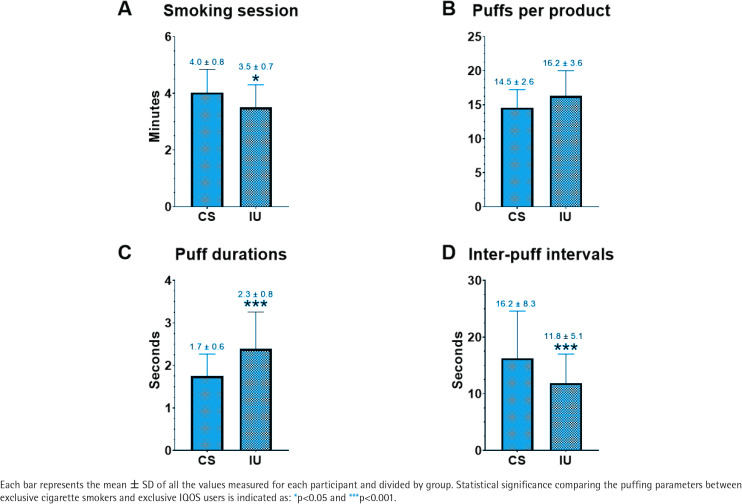
Puffing parameters evaluated in exclusive cigarette smokers (N=25) and exclusive IQOS users (N=26) included in the observational human study: A) Total length of the smoking session (minutes); B) Total number of puffs taken from each product; C: Puff durations (seconds); and D: Inter-puff intervals (seconds)

No significant differences in the number of puffs taken were detected between cigarette smokers and IQOS users (p>0.05) (Figure 2B). On the other hand, the smoking session of IQOS users was found to be substantially shorter than that of cigarette smokers (p<0.05), as they took longer and more frequent puffs than cigarette smokers (p<0.001) (Figures 2C and 2D).

## DISCUSSION

### Impact of cigarette and IQOS use on cardiovascular functionality

In this study, a significant increase in heart rate in cigarette smokers and IQOS users upon product use was detected, and substantial differences in cardiovascular parameters of cigarette smokers and IQOS users were compared with non-smokers. On the other hand, no significant differences in blood pressure and heart rate were measured between cigarette smokers and IQOS users, suggesting analogous short-term increases in blood pressure and heart rate upon cigarette and IQOS use.

Notably, industry-funded studies compared the impact of combustible cigarettes and IQOS use on cardiovascular parameters in the context of intervention studies, in which cigarette smokers were asked to use IQOS for three months^20-22^. The outcomes of these studies reported amelioration in the plasma levels of several markers related to cardiovascular risk, platelet activation, endothelial function, oxidative stress, metabolic syndrome, and lipid metabolism in cigarette smokers switching to IQOS^20-22^. However, two independent studies reported improvement of only four out of 13, and only one out of 24 biomarkers of harm in cigarette smokers switching to IQOS^4,23^. Furthermore, other industry-independent investigations described impairment of various cardiovascular parameters (left and right ventricle global longitudinal strain, flow-mediated dilation, arterial stiffness, inflammation, wave reflection, blood pressure, and heart rate) in human exposure studies upon cigarette and IQOS use^12-17,19,24^.

These findings align with previous reports and further support the hypothesis that IQOS use may induce the same acute effects on the human cardiovascular system, specifically an increase in heart rate, as conventional cigarette smoking. These results on the short-term health effects of HTPs can inform policymakers on the appropriate regulation of HTPs.

Moreover, the results can inform campaigns to increase awareness among current and potential future users about the short-term variation of parameters of cardiovascular functionality upon IQOS use.

### Puffing behavior of exclusive cigarette smokers and exclusive IQOS users

Although IQOS has been on the market for about 10 years, the smoking behavior of exclusive IQOS users is unknown. As IQOS emissions are known to contain about 50% less nicotine than cigarette smoke, it has been proposed that smokers switching to IQOS (or other HTPs) will apply compensatory puffing to satisfy their nicotine cravings^25,26^. The smoking topography of HTP users has mainly been investigated in switching studies, in which regular cigarette smokers started to use HTPs. For instance, regular cigarette smokers switching to IQOS for five days were found to take puffs with larger puff volumes, whereas those who switched to the HTP commercialized by British American Tobacco (glo) took more frequent puffs, resulting in overall shorter usage sessions^27^. Similar observations were reported by industry-funded studies, in which cigarette smokers switching to IQOS or glo increased their daily product consumption and total number of puffs per product, and took longer and more frequent puffs than participants who kept smoking their favorite brand of cigarettes^28-31^.

The smoking behavior assessment led to the identification of two distinct puffing profiles for exclusive cigarette smokers and exclusive IQOS users: cigarette smokers applied shorter and less frequent puffs, whereas longer and more frequent puffs were taken by IQOS users. As the IQOS users included in the study only consumed their preferred IQOS tobacco sticks, and no regular cigarettes or electronic cigarettes, for at least six months, their smoking profile is based on established puffing habits, which they likely developed to match their desired nicotine intake, per puff and per smoking session. Moreover, cigarette smokers switching to IQOS and established single IQOS users puff more intensely than cigarette smokers, likely due to an adaptation mechanism, to satisfy their nicotine desire and ensure effective nicotine intake^27^. This has insightful implications, as the majority of HTP users worldwide are cigarette smokers becoming dual users or even poly users of various (e-)cigarettes and HTPs^32,33^.

These results show that the puffing behavior of exclusive cigarette smokers and exclusive IQOS users is substantially different, and this notion can be used by internationally recognized tobacco testing laboratories to withdraw puffs using real-life puffing topography data^25^. Furthermore, the findings could be referential for future *in vitro* or *in vivo* comparative toxicological assessments, in which cigarette and IQOS emissions can be retrieved by applying real-life puffing parameters of each group of users. This is pivotal to accurately compare the toxicological impact of cigarette and IQOS emissions, considering that using real-life puffing parameters will help to closely mirror the real-life exposure situation^34^.

### Strengths and limitations

A strength of this study resides in having conducted it in Milan, Italy, where both exclusive cigarette smokers and exclusive IQOS users could be enrolled, allowing consideration of exclusive use of each product and excluding dual/poly users. Moreover, a home-like setting was mimicked for the participants, who could consume their tobacco product alone, outdoors, and not restricted in laboratory settings. This strengthens the scientific value of the smoking behavior assessments, as users of both cigarettes and IQOS could puff their product in silence, as they would do in their normal routine. Furthermore, a control group of never smokers was recruited, whose cardiovascular parameters were measured at T0 and T1, as done for cigarette smokers and IQOS users. Also, all participants were frequency-matched and obtained a uniform and homogeneous study population, both within each study group and between study groups. Finally, it needs to be underlined that this study was conducted independently from the tobacco industry and is free from conflicts of interest, ensuring unbiased data collection, analysis, and interpretation.

This study also presents some limitations. While during the recruitment process subjects with chronic cardiovascular and pulmonary conditions were categorically excluded, eligible participants were only required to provide information regarding age, gender, BMI, alcohol consumption and intensity of use of cigarettes or IQOS, whereas no data regarding other lifestyle parameters (e.g. physical activity, diet, stress levels, and sleep quality) that may influence the cardiovascular responses observed in the participants, were collected. Future research is advised on human studies to gather information regarding as many lifestyle indices as possible, to exclude potential confounders and enhance the scientific validity of the retrieved observations. Moreover, all the cardiovascular parameters were measured only at two time points (T0 prior to tobacco product use and T1 immediately after tobacco product use), and not over time for a longer period upon product use. Consequently, the current study design did not allow for the follow-up of the variation in cardiovascular parameters over time, to measure their peak increase as well as their renormalization. Therefore, future research aimed at monitoring the cardiovascular functionality upon cigarette and IQOS use over time is advised, starting at baseline and measuring at regular intervals of time, for about 30 minutes after product consumption^35^.

Furthermore, only the short-term effects of cigarette and IQOS use on cardiovascular function were assessed, but it is crucial to compare their long-term impacts to determine whether chronic IQOS use may lead to coronary heart disease or other cardiovascular disorders. Finally, the approach to evaluate the smoking profile of cigarette smokers and IQOS users through video recordings did not allow for measuring puff volume, which is an insightful puffing parameter that was found to be increased in cigarette smokers switching to HTPs^27^. Therefore, it is advisable that future human studies aim at evaluating also the puff volume of different users, potentially using portable measuring devices, such as the portable CReSSmicro™ device, previously used to determine the puffing profile of cigarette users^36^. In addition, reliance on self-reported questionnaire data for smoking status and health conditions introduces the potential for misclassification bias. Finally, the relatively small sample size included in our study limits statistical power and generalizability.

## CONCLUSIONS

Cigarette and IQOS use induced an almost analogous increase in blood pressure and heart rate in healthy exclusive cigarette smokers and exclusive IQOS users. Exclusive IQOS users apply a more intense puffing regime compared with exclusive cigarette smokers.

## Supplementary Material



## Data Availability

The data supporting this research are available from the authors on reasonable request.

## References

[1] Centers for Disease Control and Prevention (US). How tobacco smoke causes disease: the biology and behavioral basis for smoking-attributable disease. A report of the Surgeon General. Centers for Disease Control and Prevention; 2010. Accessed May 22, 2026. https://www.ncbi.nlm.nih.gov/books/NBK53017/21452462

[2] World Health Organization. WHO report on the global tobacco epidemic 2021: addressing new and emerging products; July 27, 2021. Accessed May 22, 2026. https://www.who.int/teams/health-promotion/tobacco-control/global-tobacco-report-2021

[3] Glantz SA. Heated tobacco products: the example of IQOS. Tob Control. 2018;27(suppl 1):s1-s6. doi:10.1136/tobaccocontrol-2018-054601 30352841 PMC6252052

[4] Fried ND, Gardner JD. Heat-not-burn tobacco products: an emerging threat to cardiovascular health. Am J Physiol Heart Circ Physiol. 2020;319(6):H1234-H1239. doi:10.1152/ajpheart.00708.2020 33006919 PMC7792702

[5] Phillips B, Veljkovic E, Boué S, et al. An 8-month systems toxicology inhalation/cessation study in ApoE-/- Mice to investigate cardiovascular and respiratory exposure effects of a candidate modified risk tobacco product, THS 2.2, compared with conventional cigarettes. Toxicol Sci. 2016;151(2):462-464. doi:10.1093/toxsci/kfw062 27225756 PMC7297302

[6] Szostak J, Titz B, Schlage WK, et al. Structural, functional and molecular impact on the cardiovascular system in ApoE-/- Mice exposed to aerosol from candidate modified risk tobacco products, carbon heated tobacco product 1.2 and tobacco heating system 2.2, compared with cigarette smoke. Chem Biol Interact. 2020;315:108887. doi:10.1016/j.cbi.2019.108887 31705857

[7] Poussin C, Laurent A, Kondylis A, et al. In vitro systems toxicology-based assessment of the potential modified risk tobacco product CHTP 1.2 for vascular inflammation- and cytotoxicity-associated mechanisms promoting adhesion of monocytic cells to human coronary arterial endothelial cells. Food Chem Toxicol. 2018;120:390-406. doi:10.1016/j.fct.2018.07.025 30026091

[8] Kondo T, Nakano Y, Adachi S, Murohara T. Effects of tobacco smoking on cardiovascular disease. Circ J. 2019;83(10):1980-1985. doi:10.1253/circj.CJ-19-0323 31462607

[9] Auer R, Diethelm P, Berthet A. Heating tobacco sticks instead of combusting conventional cigarettes and future heart attacks: still smoke and risk. Circulation. 2021;144(19):1539-1542. doi:10.1161/CIRCULATIONAHA.121.056959 34748392

[10] Qiu H, Zhang H, Han DD, et al. Increased vulnerability to atrial and ventricular arrhythmias caused by different types of inhaled tobacco or marijuana products. Heart Rhythm. 2023;20(1):76-86. doi:10.1016/j.hrthm.2022.09.021 36603937 PMC10006068

[11] Sudano I. Smoking reloaded. Atherosclerosis. 2024;390:117408. doi:10.1016/j.atherosclerosis.2023.117408 38199942

[12] Belkin S, Benthien J, Axt PN, et al. Impact of heated tobacco products, e-cigarettes and cigarettes on inflammation and endothelial dysfunction. Int J Mol Sci. 2023;24(11):9432. doi:10.3390/ijms24119432 37298381 PMC10253418

[13] Lyytinen G, Melnikov G, Brynedal A, et al. Use of heated tobacco products (IQOS) causes an acute increase in arterial stiffness and platelet thrombus formation. Atherosclerosis. 2024;390:117335. doi:10.1016/j.atherosclerosis.2023.117335 37872010

[14] Ioakeimidis N, Emmanouil E, Terentes-Printzios D, et al. Acute effect of heat-not-burn versus standard cigarette smoking on arterial stiffness and wave reflections in young smokers. Eur J Prev Cardiol. 2021;28(11):e9-e11. doi:10.1177/2047487320918365 32340460

[15] Franzen KF, Belkin S, Goldmann T, et al. The impact of heated tobacco products on arterial stiffness. Vasc Med. 2020;25(6):572-574. doi:10.1177/1358863X20943292 32721197

[16] Goebel I, Mohr T, Axt PN, et al. Impact of heated tobacco products, e-cigarettes and combustible cigarettes on small airways and arterial stiffness. Toxics. 2023;11(9):758. doi:10.3390/toxics11090758 37755768 PMC10535653

[17] Biondi-Zoccai G, Sciarretta S, Bullen C, et al. Acute effects of heat-not-burn, electronic vaping and traditional tobacco combustion cigarettes: the Sapienza University of Rome-Vascular assessment of proatherosclerotic effects of smoking ( SUR - VAPES ) 2 randomized trial. J Am Heart Assoc. 2019;8(6):e010455. doi:10.1161/JAHA.118.010455 30879375 PMC6475061

[18] Liu X, Lugo A, Spizzichino L, Tabuchi T, Gorini G, Gallus S. Heat-Not-Burn tobacco products are getting hot in Italy. J Epidemiol. 2018;28(5):274-275. doi:10.2188/jea.JE20180040 29657258 PMC5911679

[19] Yaman B, Akpınar O, Kemal HS, et al. Comparison of IQOS (heated tobacco) and cigarette smoking on cardiac functions by two-dimensional speckle tracking echocardiography. Toxicol Appl Pharmacol. 2021;423:115575. doi:10.1016/j.taap.2021.115575 34000265

[20] Lüdicke F, Picavet P, Baker G, et al. Effects of switching to the menthol tobacco heating system 2.2, smoking abstinence, or continued cigarette smoking on clinically relevant risk markers: a randomized, controlled, open-label, multicenter study in sequential confinement and ambulatory settings (part 2). Nicotine Tob Res. 2018;20(2):173-182. doi:10.1093/ntr/ntx028 28177498 PMC5896432

[21] Haziza C, de La Bourdonnaye G, Donelli A, et al. Reduction in exposure to selected harmful and potentially harmful constituents approaching those observed upon smoking abstinence in smokers switching to the menthol tobacco heating system 2.2 for 3 months (part 1). Nicotine Tob Res. 2020;22(4):539-548. doi:10.1093/ntr/ntz013 30722062 PMC7164581

[22] Haziza C, de La Bourdonnaye G, Donelli A, et al. Favorable changes in biomarkers of potential harm to reduce the adverse health effects of smoking in smokers switching to the menthol tobacco heating system 2.2 for 3 months (part 2). Nicotine Tob Res. 2020;22(4):549-559. doi:10.1093/ntr/ntz084 31125079 PMC7164580

[23] Glantz SA. PMI’s own in vivo clinical data on biomarkers of potential harm in Americans show that IQOS is not detectably different from conventional cigarettes. Tob Control. 2018;27(Suppl 1):s9-s12. doi:10.1136/tobaccocontrol-2018-054413 30131374 PMC6202159

[24] Nabavizadeh P, Liu J, Havel CM, et al. Vascular endothelial function is impaired by aerosol from a single IQOS HeatStick to the same extent as by cigarette smoke. Tob Control. 2018;27(Suppl 1):s13-s19. doi:10.1136/tobaccocontrol-2018-054325 30206183 PMC6202192

[25] Davigo M, Klerx WNM, van Schooten FJ, Opperhuizen A, Remels AHV, Talhout R. Impact of more intense smoking parameters and flavor variety on toxicant levels in emissions of a heated tobacco product. Nicotine Tob Res. 2024;26(5):571-579. doi:10.1093/ntr/ntad238 38035623 PMC11033558

[26] Vukas J, Mallock-Ohnesorg N, Rüther T, et al. Two different heated tobacco products vs. cigarettes: comparison of nicotine delivery and subjective effects in experienced users. Toxics. 2023;11(6):525. doi:10.3390/toxics11060525 37368625 PMC10301154

[27] Jones J, Slayford S, Gray A, Brick K, Prasad K, Proctor C. A cross-category puffing topography, mouth level exposure and consumption study among Italian users of tobacco and nicotine products. Sci Rep. 2020;10(1):12. doi:10.1038/s41598-019-55410-5 31913299 PMC6949288

[28] Lüdicke F, Baker G, Magnette J, Picavet P, Weitkunat R. Reduced exposure to harmful and potentially harmful smoke constituents with the tobacco heating system 2.1. Nicotine Tob Res. 2017;19(2):168-175. doi:10.1093/ntr/ntw164 27613951 PMC5234364

[29] Haziza C, de La Bourdonnaye G, Merlet S, et al. Assessment of the reduction in levels of exposure to harmful and potentially harmful constituents in Japanese subjects using a novel tobacco heating system compared with conventional cigarettes and smoking abstinence: a randomized controlled study in confinement. Regul Toxicol Pharmacol. 2016;81:489-499. doi:10.1016/j.yrtph.2016.09.014 27693654

[30] Haziza C, de La Bourdonnaye G, Skiada D, et al. Evaluation of the tobacco heating system 2.2. part 8: 5-day randomized reduced exposure clinical study in Poland. Regul Toxicol Pharmacol. 2016;81(Suppl 2):s139-s150. doi:10.1016/j.yrtph.2016.11.003 27816672

[31] Lüdicke F, Picavet P, Baker G, et al. Effects of switching to the tobacco heating system 2.2 menthol, smoking abstinence, or continued cigarette smoking on biomarkers of exposure: a randomized, controlled, open-label, multicenter study in sequential confinement and ambulatory settings (part 1). Nicotine Tob Res. 2018;20(2):161-172. doi:10.1093/ntr/ntw287 28177489 PMC5896533

[32] Sugiyama T, Tabuchi T. Use of Multiple tobacco and tobacco-like products including heated tobacco and e-cigarettes in Japan: a cross-sectional assessment of the 2017 JASTIS study. Int J Environ Res Public Health. 2020;17(6):2161. doi:10.3390/ijerph17062161 32213924 PMC7143444

[33] Sun T, Anandan A, Lim CCW, et al. Global prevalence of heated tobacco product use, 2015-22: a systematic review and meta-analysis. Addiction. 2023;118(8):1430-1444. doi:10.1111/add.16199 37005862

[34] Ardati O, Adeniji A, El Hage R, et al. Impact of smoking intensity and device cleaning on IQOS emissions: comparison with an array of cigarettes. Tob Control. 2024;33(4):449-456. doi:10.1136/tc-2022-057802 36609493 PMC10323035

[35] Dimitriadis K, Narkiewicz K, Leontsinis I, et al. Acute effects of electronic and tobacco cigarette smoking on sympathetic nerve activity and blood pressure in humans. Int J Environ Res Public Health. 2022;19(6):3237. doi:10.3390/ijerph19063237 35328926 PMC8952787

[36] Pauwels CGGM, Boots AW, Visser WF, et al. Characteristic human individual puffing profiles can generate more TNCO than ISO and health Canada regimes on smoking machine when the same brand is smoked. Int J Environ Res Public Health. 2020;17(9):3225. doi:10.3390/ijerph17093225 32384697 PMC7246490

