# Supplementary material for: Short-term cardiovascular effects and puffing behavior of exclusive heated tobacco product (HTP) users: A quasi-experimental study
Source: Tob Induc Dis. 2026 Jul 20;24:10.18332/tid/222366. doi: 10.18332/tid/222366 (PMC13401254; doi:10.18332/tid/222366)
Supplement: Supplementary file 1 [file TID-24-119-s1.pdf]

TID
Tobacco Induced Diseases

Supplementary file

© 2026 Davigo M. et al.

DOI:
10.18332/tid/222366

The content has been provided by the author(s) and has not been reviewed, verified, or endorsed by European Publishing. It may not have undergone peer review. The views, opinions, and recommendations expressed are solely those of the author(s) and do not necessarily reflect the position of European Publishing. European Publishing accepts no responsibility or liability for any consequences arising from the use of, or reliance on, this content.

Questionario - Studio osservazionale sugli effetti a breve termine dell’utilizzo di IQOS

SEZIONE A - Dati di classificazione demografica

* 1. Nome

* 2. Contatto (indirizzo email e/o numero di telefono) fornito durante la registrazione al presente studio

* 3. Genere

- ◯ Maschio
- ◯ Femmina

* 4. Età (anni compiuti)

Questionario - Studio osservazionale sugli effetti a breve termine dell’utilizzo di IQOS

SEZIONE B – Utilizzo di prodotti del tabacco e altri prodotti

* 5. Hai fumato almeno 100 sigarette tradizionali (considerando anche le sigarette rollate a mano ma non considerando sigarette elettroniche e prodotti a tabacco riscaldato) nel corso di tutta la tua vita?

- ◯ Sì
- ◯ No

Questionario - Studio osservazionale sugli effetti a breve termine dell’utilizzo di IQOS

* 6. Fumi sigarette attualmente?

- ◯ Sì
- ◯ No

Questionario - Studio osservazionale sugli effetti a breve termine dell’utilizzo di IQOS

* 7. In media, quante sigarette fumi al giorno, considerando anche quelle rollate a mano?

Questionario - Studio osservazionale sugli effetti a breve termine dell’utilizzo di IQOS

* 8. Usi (o hai usato) la sigaretta elettronica (comprese quelle con sali o cristalli di nicotina come Juul o My Blue, e quelle monouso come Puff Bars)?

- Mai usata
- L’ho provata solo 1-2 volte
- L’ho usata in passato (ma non negli ultimi 30 giorni)
- La uso occasionalmente
- La uso regolarmente (consumo giornaliero)

* 9. Usi IQOS (dispositivo a tabacco riscaldato) con stick HEETS?

- Mai usata
- L’ho provata solo 1-2 volte
- L’ho usata in passato (ma non negli ultimi 30 giorni)
- La uso occasionalmente
- La uso regolarmente (consumo giornaliero)

Questionario - Studio osservazionale sugli effetti a breve termine dell’utilizzo di IQOS

* 10. In media, quante HEETS (stick di tabacco riscaldato) fumi al giorno?

Questionario - Studio osservazionale sugli effetti a breve termine dell’utilizzo di IQOS

* 11. Usi IQOS ILUMA (un dispositivo a tabacco riscaldato) con stick TEREA?

- ◯ Mai usata
- ◯ L'ho provata solo 1-2 volte
- ◯ L'ho usata in passato (ma non negli ultimi 30 giorni)
- ◯ La uso occasionalmente
- ◯ La uso regolarmente (consumo giornaliero)

Questionario - Studio osservazionale sugli effetti a breve termine dell'utilizzo di IQOS

* 12. In media, quante TEREA (stick di tabacco riscaldato) fumi al giorno?

Questionario - Studio osservazionale sugli effetti a breve termine dell'utilizzo di IQOS

* 13. Usi altri dispositivi a tabacco riscaldato (glo, Ploom)?

- ◯ Mai usati
- ◯ Li ho provati solo 1-2 volte
- ◯ Li ho usati in passato (ma non negli ultimi 30 giorni)
- ◯ Li uso occasionalmente
- ◯ Li uso regolarmente (consumo giornaliero)

Questionario - Studio osservazionale sugli effetti a breve termine dell'utilizzo di IQOS

SEZIONE C – Stili di vita

* 14. In media, quante unità alcoliche consumi attualmente? Fai riferimento all’unità alcolica, che corrisponde a 1 lattina di birra (330 ml) o 1 bicchiere di vino (125 ml) o 1 aperitivo (80 ml) o 1 superalcolico (40 ml).

- ◯ Nessuna
- ◯ 1-3 unità alcoliche al mese
- ◯ 1-6 unità alcoliche alla settimana
- ◯ 1 unità alcolica al giorno
- ◯ 2 unità alcoliche al giorno
- ◯ 3 unità alcoliche al giorno
- ◯ 4 unità alcoliche al giorno
- ◯ 5 unità alcoliche al giorno
- ◯ 6 o più unità alcoliche al giorno

Questionario - Studio osservazionale sugli effetti a breve termine dell’utilizzo di IQOS

Informativa al trattamento dei dati personali

* 15. Informativa e consenso per la raccolta ed il trattamento dei dati personali

- ☐ Ho letto l'informativa sulla privacy e acconsento al trattamento dei miei dati personali

Supplementary Table 1: Inclusion and exclusion criteria for each group of the study.

	**Study group**		
	**Never Smokers (group 1)**	**Conventional cigarette users (group 2)**	**IQOS users (group 3)**
Age	≥18 years old	≥18 years old	≥18 years old
Gender	Any	Any	Any
Body mass index (BMI)	Normal weight (BMI: 18.5 – 25.0 kg/m2)	Normal weight (BMI: 18.5 – 25.0 kg/m2)	Normal weight (BMI: 18.5 – 25.0 kg/m2)
Conventional cigarette use	Never use	Current use: ≥1 year of smoking, ≥4 cigarettes per day	No daily use over the last 6 months
IQOS use	Never use	No daily use over the last 6 months	Current use: ≥ 6 months of use, ≥ 4 sticks (either HEETS or TEREA) per day
Electronic cigarette use	Never use	No daily use over the last 6 months	No daily use over the last 6 months
Health status	None of the following: cancer, smoking-related respiratory diseases (e.g., Chronic obstructive pulmonary disease), metabolic diseases, cardiovascular risk factors or cardiovascular disease (e.g., high blood pressure, high heart rate), aspects of metabolic syndrome, and use of medication associated with cardiovascular disease (e.g., blood pressure lowering medication, β-blockers, cholesterol-lowering medication)	None of the following: cancer, smoking-related respiratory diseases (e.g., Chronic obstructive pulmonary disease), metabolic diseases, cardiovascular risk factors or cardiovascular disease (e.g., high blood pressure, high heart rate), aspects of metabolic syndrome, and use of medication associated with cardiovascular disease (e.g., blood pressure lowering medication, β-blockers, cholesterol-lowering medication)	None of the following: cancer, smoking-related respiratory diseases (e.g., Chronic obstructive pulmonary disease), metabolic diseases, cardiovascular risk factors or cardiovascular disease (e.g., high blood pressure, high heart rate), aspects of metabolic syndrome, and use of medication associated with cardiovascular disease (e.g., blood pressure lowering medication, β-blockers, cholesterol-lowering medication)
Pregnancy and lactation	No	No	No
